# Transplanters drive CARs to the clinic by brewing ICE-T: the Moffitt roadmap

**DOI:** 10.1186/s40425-017-0265-y

**Published:** 2017-07-18

**Authors:** Frederick L. Locke, Claudio Anasetti, for the Moffitt Immunotherapy Working Group and the Immune Cell Therapy (ICE-T) Program

**Affiliations:** 1 Department of Blood and Marrow Transplant and Cellular Immunotherapy, Moffitt Cancer Center, 12902 Magnolia Dr. FOB-3, Tampa, FL 33612 USA; 2 Department of Oncologic Sciences, University of South Florida, Tampa, USA; 3 Immunology Program, Moffitt Cancer Center, Tampa, USA

**Keywords:** Chimeric antigen receptor (CAR) therapy, Cytotoxic T lymphocytes, Cell therapy, T cell receptor (TCR) therapy, Tumor infiltrating lymphocyte (TIL) therapy, Cytokine release syndrome

## Abstract

Recent single institution clinical trial successes with anti-CD19 Chimeric Antigen Receptor (CAR) T cell therapy for B cell malignancies attracted significant attention from industry. Our center sought to rapidly and safely bring industry sponsored pivotal trials to our patients and to prepare for additional cell therapy trials in solid and liquid tumors from both industry and our own investigators. We implemented a collaborative cross departmental program to provide clinical care and trial coordination for immune cell therapies. The Moffitt Immune Cell Therapy (ICE-T) program oversees and administers not only CAR T cell therapy for hematologic malignancies, but TIL and TCR therapy for solid tumor patients. Disease specific experts maintain oversight as principal investigators of these key trials yet the coordination and clinical care is centralized to leverage the expertise and infrastructure of our already robust Blood and Marrow Transplantation program.

## Commentary

Single institutions reported response rates of 50–90% in refractory B cell malignancies to anti-CD19 chimeric antigen receptor (CAR) T cells, and 80% in advanced myeloma to T cell Receptor (TCR) T cells against cancer antigens [1–5]. Multiple pharmaceutical companies licensed anti-CD19 CAR and TCR constructs and initiated pivotal multi-center clinical trials aiming to prove clinical benefit and secure FDA approved indications [6, 7]. The safety profile of CAR T cells remains overall favorable. However, complexity including cell collection, cryopreservation and shipments, chemotherapy delivery, care after cell infusion, and management of side effects including pancytopenia, cytokine release syndrome and neurotoxicity, make careful implementation of this therapy paramount [4]. Here, as a roadmap for others, we report how our Blood and Marrow Transplant (BMT) team was instrumental in creating an interdisciplinary program and successfully implemented TCR and CAR T cell therapy for both liquid and solid tumors.

First, we broke down disease-specific silos by assembling an interdisciplinary Immunotherapy Working Group consisting of transplant and non-transplant physician hematologists, non-physician immunologists, and solid tumor oncologists. The Immunotherapy Working Group identified areas of strength to exploit and weaknesses to address, and selected TCR, CAR T cell, and other cell therapy clinical trials with the highest scientific merit.

Second, we elected to utilize BMT professional staff, and inpatient and outpatient BMT units for the clinical care of solid tumor and liquid tumor immune cell therapy trial patients. With >400 hematopoietic transplants annually, personnel was skilled in apheresis, cell handling, ambulatory and inpatient care. Our BMT department and cell therapies facility maintain accreditation for Hematopoietic Cellular Therapy by the Foundation for the Accreditation of Cellular Therapy (FACT), therefore the processes, documentation and oversight required for distribution, receipt and administration of cellular therapies was in place. However, we recognized the risks of merely adding cellular immunotherapy patients to the BMT clinical service without planning for tailored standard operating procedures and education of Faculty and staff: prior to involvement in these industry sponsored clinical trials we had no experience with cytokine release syndrome and neurological toxicities caused by CARs. Both toxicities can be life threatening and careful groundwork is essential.

Third, we developed the Immune Cell Therapy (ICE-T) service to gain expertise caring for patients treated with TCR and CAR T cells, or other immunotherapies with high risk for acute toxicities. A core group of hematologists and medical oncologists from multiple departments volunteered responsibility for the care of cell therapy patients, 7 days per week. Didactic sessions and written resources were shared with all involved physicians, advanced practice professionals, nurses and ancillary staff. As the census grew, training and exposure to these patients became broader. Nurse coordinators were engaged to educate patients about the treatment process, act as liaison between the ICE-T service and the referring physicians, and provide nursing expertise before and after therapy. Monthly multidisciplinary ICE-T meetings were instituted to disseminate knowledge about unique toxicities and create standard operating procedures defining care guidelines for cellular therapy patients of all tumor types. The Moffitt ICE-T service provides complete inpatient (cell infusion and toxicity management) and outpatient (apheresis, conditioning chemotherapy, and post discharge care) medical care. At day +30 following cellular therapy, those patients no longer requiring intensive outpatient care return to their referring medical oncologist.

Fourth, we created a dedicated Clinical Research team for ICE-T separate from BMT, Malignant Hematology, Thoracic, Sarcoma, etc. Liquid and solid tumor cellular therapy trials necessitate a high degree of logistical coordination between patients, investigators, manufacturing facilities, trial medical directors, and clinical research monitors. The FDA and study sponsors require real-time data collection for these patients. Clinical trial coordinators and research data specialists require higher effort for each patient as compared to trials of cyclical chemotherapy infusions or oral drug regimens. In addition, a regulatory affairs office facilitates successful FDA Investigational New Drug (IND) applications for Moffitt’s investigator-initiated cell therapy product clinical trials.

The ICE-T service and clinical research program have allowed us to quickly and safely open multiple industry sponsored cellular therapy trials for hematologic and solid tumors; supported investigator efforts implementing grant funded, investigator-initiated clinical trials; gained recognition by the National Cancer Institute (P30 CA076292 and P30 CA076292-18S4); and expedited the design and implementation of processes and oversight acceptable for the recently established FACT Standards for the use of Immune Effector Cells [8]. Initial analyses demonstrated satisfactory reimbursement for standard care costs associated with cellular immunotherapy. With pivotal trial results now available, several pharmaceutical companies anticipate FDA approved indications for anti-CD19 CAR T cell therapy in 2017 [9, 10]. Other cellular immunotherapies such as TCR T cells, tumor-infiltrating lymphocytes, and tumor vaccines are attracting attention from industry signaling increased multicenter trials and therapeutic indications. Identifying the resources needed during the clinical trial period, along with anticipation of volumes, has prepared us for great expansion of the infrastructures for cell therapy delivery.
